# Applicability of Whole Blood Monocyte Activation Test for Endotoxin Activity Assessment in Hydroxyapatite-Based Ceramics

**DOI:** 10.3390/bioengineering13030319

**Published:** 2026-03-11

**Authors:** Janaina Spoladore, Carolina Barbara Nogueira de Oliveira, Joice Correa da Silva, Elena Mavropoulos Tude, Carlos Fernando Mourão, Gutemberg Gomes Alves

**Affiliations:** 1 Post-Graduation Program in Science and Biotechnology, Institute of Biology, Fluminense Federal University, Niteroi 24210-201, Brazil; janspoladore@gmail.com (J.S.); joicecorrea@id.uff.br (J.C.d.S.); 2 Brazilian Center for Validation of Alternative Methods (BraCVAM), Instituto de Ciência e Tecnologia em Biomodelos (ICTB), FIOCRUZ, Rio de Janeiro 21040-360, Brazil; carolina.deoliveira@fiocruz.br; 3 Brazilian Center for Physics Research, Rio de Janeiro 22290-180, Brazil; elena@cbpf.br; 4 Department of Clinical and Translational Research, Tufts University School of Dental Medicine, Boston, MA 02111, USA; 5 Clinical Research Unit, Antônio Pedro University Hospital, Fluminense Federal University, Niteroi 24033-900, Brazil; gutemberg_alves@id.uff.br

**Keywords:** hydroxyapatite, ceramics, endotoxins, lipopolysaccharides, cytokines, blood

## Abstract

Hydroxyapatite-based ceramics are widely used in dental bioengineering, yet the reliable assessment of endotoxin activity in solid porous materials remains challenging. This study evaluated the applicability of a whole blood Monocyte Activation Test (MAT) to a hydroxyapatite ceramic relevant to dental applications. Two endotoxin association strategies (immersion and dropwise) were compared, followed by nonlinear modelling of cytokine dose–response curves using four-parameter logistic (4PL) regression and spike-recovery analysis to assess potential material interference. Immersion-based spiking produced reproducible, concentration-dependent cytokine responses, whereas dropwise application resulted in minimal functional recovery. IL-1β, IL-6, and TNF-α displayed sigmoidal dose–response profiles with high goodness-of-fit values (R^2^ ≥ 0.93). Spike recovery remained within the 50–200% acceptance range for most concentrations, with IL-6 showing the most consistent analytical performance. TNF-α exhibited signal saturation at higher endotoxin levels, limiting its dynamic range. Multiplex cytokine profiling confirmed that classical MAT readouts were among the most strongly induced mediators and that hydroxyapatite did not trigger baseline inflammatory activation. These findings demonstrate that whole blood MAT can be reliably applied to hydroxyapatite-based dental ceramics when immersion-based endotoxin association and material-specific methodological optimization are employed.

## 1. Introduction

Hydroxyapatite-based ceramics are widely used in dental bioengineering as bone graft substitutes, implant coatings, and regenerative scaffolds due to their chemical similarity to mineralized tissue and well-documented osteoconductive properties [1]. Their bioactivity, surface reactivity, and structural tunability make them attractive platforms for applications ranging from alveolar ridge augmentation to guided bone regeneration and implant surface modification [2] and have further supported the development of advanced composite and polymer-coated hydroxyapatite systems for enhanced biomedical performance [3]. As the clinical use of synthetic calcium phosphate ceramics continues to expand, ensuring their biological safety becomes increasingly critical, particularly for materials intended for implantation or prolonged tissue contact [4].

Among safety concerns, endotoxin contamination represents a significant and often underestimated risk [5]. Endotoxins, primarily lipopolysaccharides (LPS) derived from Gram-negative bacteria, may be introduced during raw material processing, handling, or manufacturing steps. Due to the high surface area and adsorption capacity of porous ceramics, endotoxin molecules may associate with material surfaces, potentially altering their biological availability while retaining inflammatory activity [6]. In a clinical setting, even low levels of biologically active endotoxin may trigger local inflammatory responses that compromise tissue integration and regenerative outcomes [7]. Therefore, the reliable detection of endotoxin activity in hydroxyapatite-based dental biomaterials is not only a regulatory requirement but also a critical component of translational bioengineering.

The detection of endotoxins in biomedical products has traditionally relied on limulus amebocyte lysate (LAL)-based assays [8]. Although widely adopted, these methods primarily detect endotoxin through a biochemical cascade rather than through functional human immune activation [9]. In addition, LAL assays may be susceptible to interference when applied to complex or solid materials, particularly those with high adsorption capacity, variable surface chemistry, or particulate characteristics [10]. In such contexts, endotoxin molecules may bind to the material surface, potentially affecting assay sensitivity or leading to underestimation of biologically relevant activity.

The Monocyte Activation Test (MAT) has emerged as a human cell-based alternative that measures cytokine release following exposure to pyrogens. By assessing functional immune activation rather than solely detecting endotoxin presence, MAT provides a biologically relevant readout of inflammatory potential [11]. While MAT has been extensively evaluated for pharmaceutical products and liquid formulations, its application to solid biomaterials remains less standardized [12]. Materials with porous architecture and reactive surfaces, such as hydroxyapatite ceramics, present specific methodological challenges, including the mode of endotoxin association, potential matrix interference, and the selection of appropriate cytokine readouts [13]. For hydroxyapatite-based dental ceramics, these considerations are particularly relevant. The intrinsic physicochemical properties that confer osteoconductivity and surface bioactivity may also influence endotoxin adsorption and immune recognition [14]. Consequently, the implementation of MAT in this context requires systematic evaluation of assay parameters to ensure reliable detection of endotoxin activity without material-induced distortion of cytokine responses. In addition, the selection of the biological matrix and exposure format is particularly relevant for porous calcium phosphate ceramics. The recent literature has emphasized that MAT performance in medical devices depends not only on cytokine selection but also on the biological matrix employed (e.g., fresh whole blood, cryopreserved blood, or monocytic cell lines) and on whether testing is conducted by direct material contact or via eluate [12]. Because hydroxyapatite exhibits high surface area and adsorption capacity, endotoxin molecules may remain surface-associated and may not be fully recovered by extraction-based approaches. In this context, a whole blood MAT combined with direct contact exposure provides a biologically relevant advantage, as it enables immune cells to interact directly with surface-bound pyrogens and reduces the risk of underestimating functional endotoxin activity in ceramic matrices [13].

Despite increasing interest in applying MAT to solid biomaterials, there remains limited systematic evaluation of its performance in ceramic systems used in dental applications [12]. Few studies have addressed how different endotoxin association strategies may influence assay reliability in porous hydroxyapatite substrates, or how quantitative modelling of cytokine responses can support interpretation of functional endotoxin activity in whole blood-based MAT configurations [15]. Moreover, the comparative performance of commonly used MAT cytokine readouts in the specific context of hydroxyapatite-based ceramics has not been comprehensively characterized particularly when direct material–blood contact approaches are employed.

To address these gaps, this study evaluates the applicability of a whole blood MAT platform to a hydroxyapatite-based ceramic system relevant to dental bioengineering. We systematically investigated methodological parameters influencing assay performance, namely: (i) comparison of endotoxin association approaches, (ii) nonlinear modelling of dose–response behaviour via four-parameter logistic regression, (iii) spike-recovery analysis for potential material interference, and (iv) extended cytokine profiling by multiplex analysis. Ultimately, this work aims to establish a structured framework for endotoxin activity assessment in hydroxyapatite-based dental ceramics.

## 2. Materials and Methods

### 2.1. Hydroxyapatite Discs

Surgical-grade hydroxyapatite (HA) discs were used as the test biomaterial in this study. The discs were supplied by the Brazilian Center for Physical Research (Rio de Janeiro, Brazil) and produced according to a previously described protocol. Briefly, stoichiometric hydroxyapatite powder was pressed into cylindrical discs under uniaxial compression and subsequently sintered at high temperature (1100 °C) to obtain dense ceramic structures, as previously described [16]. The HA discs were cylindrical, with nominal dimensions of 12 mm in diameter and 2 mm in thickness.

All discs used in the experiments originated from the same production batch to minimize variability related to composition or processing. Prior to experimental use, the discs were handled exclusively under aseptic conditions and stored in sterile containers at room temperature until further processing. No additional surface modification or chemical treatment was applied before the depyrogenation and endotoxin spiking procedures described below.

### 2.2. Material Characterization

To confirm the structural identity and crystalline phase of the hydroxyapatite discs used in the biological assays, the material was characterized by Fourier transform infrared spectroscopy (FTIR) and X-ray diffraction (XRD) prior to biological testing. FTIR analysis was performed to identify the characteristic vibrational bands associated with phosphate (PO_4_^3−^) and hydroxyl (OH^−^) groups typical of hydroxyapatite. Spectra were acquired in the mid-infrared region using a Fourier transform IR Prestige-21 spectrophotometer (Shimadzu, Kyoto, Japan), and the resulting spectra were analyzed qualitatively for the presence of bands consistent with stoichiometric hydroxyapatite. No quantitative phase analysis (QPA) was performed, as the objective of this characterization step was limited to qualitative confirmation of phase identity and the absence of detectable secondary crystalline phases prior to biological evaluation.

X-ray diffraction analysis was conducted to assess the crystalline structure and phase composition of the discs. Diffraction patterns were obtained using an X-ray diffractometer (HZG4, Zeiss, Oberkochen, Germany) over a 2θ range of 10–60°, and the observed diffraction peaks were compared the standard reference pattern for stoichiometric hydroxyapatite (ICDD PDF No. 09-0432).

### 2.3. In Vitro Cytocompatibility

To exclude overt cytotoxicity of the hydroxyapatite used in the cytokine assessments, apoptosis and necrosis were evaluated in human monocyte-derived macrophages after exposure to material extracts. The extract-based approach was selected to evaluate the potential release of soluble and leachable substances from the ceramic discs, minimizing physical surface interference and allowing standardized assessment of material-derived biological effects. Peripheral blood was obtained from a single healthy donor collected in heparinized tubes. Peripheral blood mononuclear cells (PBMCs) were isolated by density gradient centrifugation (Histopaque^®^ 1083, Sigma-Aldrich) at 460× *g* for 28 min at 25 °C. Mononuclear cells collected from the interphase were seeded onto sterile glass coverslips in 24-well plates at a density of 2 × 10^6^ cells per well and cultured in RPMI 1640 medium (Gibco, Grand Island, NY, USA) supplemented with 10% fetal bovine serum at 37 °C in 5% CO_2_. Medium was replaced every 2 days to allow monocyte adherence and differentiation into macrophage-like cells over 6 days. Material extracts were prepared according to ISO 10993-12 guidelines [17] by incubating HA discs in RPMI medium at a surface area-to-volume ratio of 6 cm^2^/mL at 37 °C for 24 h. On day 6, culture medium was replaced with the respective extracts and cells were exposed for 24 h. Latex extracts (at 200 mg/mL in RPMI) and 0.25 µM staurosporine (Merck, Rahway, NJ, USA) were used as positive controls for necrosis and apoptosis, respectively. After exposure, cells were stained using Annexin V-FITC and propidium iodide (Aposcreen Annexin V-FITC, R&D Systems, Minneapolis, MN, USA) according to the manufacturer’s instructions. Fluorescence analysis was performed using an inverted fluorescence microscope (Axio A1, Zeiss, Oberkochen, Germany). For each coverslip, the first 100 cells observed were classified as viable (Annexin V−/PI−), early apoptotic (Annexin V+/PI−), late apoptotic (Annexin V+/PI+), or necrotic (Annexin V−/PI+). The experiment was independently repeated twice with quintuplicate coverslips per condition.

### 2.4. Depyrogenation of Hydroxyapatite Discs

In order to eliminate residual endotoxins potentially present on the surface of the hydroxyapatite discs prior to experimental contamination, all samples were subjected to a thermal depyrogenation procedure. The discs were placed in heat-resistant containers and treated by dry heat at 250 °C for 30 min in a laboratory oven. This procedure was selected in accordance with pharmacopeial dry-heat depyrogenation recommendations for endotoxin inactivation (European Pharmacopoeia 2.6.14), which recognize such heat exposure as effective for endotoxin destruction in solid surfaces such as glassware [18]. The adequacy of the procedure was functionally supported by low baseline cytokine levels in blank control conditions during subsequent MAT analysis. After heat treatment, the discs were allowed to cool to room temperature under controlled conditions and were subsequently handled using sterile instruments to avoid recontamination. Depyrogenated discs were stored in sterile containers until use in the endotoxin spiking and whole blood exposure experiments described below.

### 2.5. Endotoxin Preparation and LPS Standard Curve

Lipopolysaccharide (LPS) from Escherichia coli (serotype O55:B5, Sigma-Aldrich, Steinheim, Germany) was used as the endotoxin stimulus in all experiments. A 1 ng/mL stock solution was prepared according to the manufacturer’s instructions using sterile, pyrogen-free aqueous solution and stored under conditions recommended by the supplier to preserve endotoxin activity.

Working solutions were freshly prepared on the day of each experiment by serial dilution of the stock solution in sterile, endotoxin-free aqueous solution adjusted to physiological ionic strength. LPS concentrations were expressed in endotoxin units (EU/mL), and the working range was selected based on preliminary titration experiments to encompass the dynamic response window of whole blood MAT, spanning 0.25 to 5 EU/mL. The LPS standard curve served as a positive control for inflammatory activation and was used as a reference to compare cytokine responses induced by endotoxin-contaminated hydroxyapatite discs in subsequent experiments.

### 2.6. Endotoxin Spiking of Hydroxyapatite Discs

Depyrogenated hydroxyapatite discs were contaminated with defined amounts of endotoxin using two different spiking approaches. Both immersion-based and dropwise endotoxin spiking methods have been previously reported for simulating endotoxin contamination on biomaterial surfaces [12]. Therefore, both approaches were evaluated in the present study.

For immersion-based spiking, individual discs were fully submerged in 1 mL of sterile endotoxin solutions containing LPS at concentrations corresponding to final nominal loads of 0.25, 0.5, 1.0, 2.5, and 5.0 EU per disc. Immersion was performed under static conditions at 37 °C for 1 h to allow adsorption of endotoxin onto the hydroxyapatite surface. After incubation, discs were removed from the solution using sterile forceps and allowed to dry under aseptic conditions in a laminar flow cabinet for approximately 30 min prior to whole blood exposure.

For dropwise spiking, 100 µL of LPS solution at the desired concentration (adjusted to deliver the same nominal endotoxin loads of 0.25–5.0 EU per disc) was directly applied onto the upper surface of each disc and allowed to dry at room temperature under sterile conditions for 30 min. Following drying, the discs were processed in the same manner as immersion-spiked samples.

All spiked discs were used immediately after drying or stored for a short period (up to 4 h) under sterile conditions at room temperature prior to exposure to whole blood. The effectiveness of the two spiking approaches was experimentally compared based on cytokine release and spike-recovery performance, as described in the Section 3.

### 2.7. Human Blood Collection

Peripheral human blood was obtained from healthy adult volunteers after written informed consent. All procedures involving human participants were conducted in accordance with the ethical standards of the institutional and national research committee and with the 1964 Declaration of Helsinki and its later amendments. The study protocol was approved by the Research Ethics Committee of the Faculty of Medicine, Fluminense Federal University (CAAE 57824616.0.1001.5243, approval 1.772.226). Blood was collected by venipuncture into sterile tubes containing unfractionated heparin as anticoagulant and processed immediately after collection. Only donors without acute or chronic inflammatory conditions, recent infections, or current use of anti-inflammatory, immunosuppressive, or immunomodulatory medications were included. A total of five healthy adult donors participated in the study.

To minimize inter-individual variability in cytokine responses, whole blood samples from individual donors were combined to form pooled whole blood preparations prior to experimental use. Pooling was performed by gently mixing equal volumes of whole blood from each donor under sterile conditions. The pooled blood was subsequently diluted 1:10 using sterile, endotoxin-free aqueous solution adjusted to physiological ionic strength.

Donor-specific cytokine responses were not evaluated individually prior to pooling, as the study objective was to assess assay performance under a standardized pooled-blood configuration rather than to characterize inter-individual variability. Formal intra-pool variability analysis across experimental runs was not performed; however, consistency of dose–response behaviour and goodness-of-fit parameters across independent experiments supported the analytical stability of the pooled system. All pooled blood preparations were used fresh on the day of collection and were maintained at controlled room temperature until use in whole blood exposure experiments. The total time between blood collection and experimental exposure did not exceed 8 h.

### 2.8. Whole Blood Exposure to Contaminated Discs

Following endotoxin spiking, hydroxyapatite discs were exposed to pooled human whole blood. Immediately after blood collection and verification of basic hematological parameters, individual samples were combined under sterile conditions to obtain pooled whole blood. For whole blood exposure, each endotoxin-spiked or control hydroxyapatite disc was placed individually into sterile Falcon tubes. Sterile, endotoxin-free 5% glucose solutions were used as an isotonic diluent compatible with whole blood MAT configurations, minimizing interference from culture medium components and ensuring osmotic stability during incubation, in accordance with pharmacopoeial MAT principles [19]. To each tube, 1 mL of 5% glucose solution and 100 µL of pooled whole blood were added. Discs contaminated with different endotoxin concentrations (0.25, 0.5, 1, 2.5, and 5 EU) as well as non-spiked control discs were tested under identical conditions.

In parallel, an LPS reference curve was prepared by incubating pooled whole blood directly with soluble LPS at the same endotoxin concentrations (0.25, 0.5, 1, 2.5, and 5 EU) in the absence of hydroxyapatite discs. These samples contained 1 mL of 5% glucose solution and 100 µL of pooled whole blood, allowing direct comparison between soluble endotoxin and endotoxin associated with the biomaterial surface.

Separate tubes were prepared for each incubation time point. Tubes designated for TNF-α quantification were incubated for 4 h at 37 °C under semi-open conditions with aluminum foil protection to prevent environmental contamination, and supernatants were collected at that time. Independent tubes prepared under identical conditions for IL-1β and IL-6 analysis were incubated overnight (approximately 16–18 h) before supernatant collection. Following incubation, samples were processed for supernatant collection and subsequent cytokine analysis as described below.

### 2.9. Cytokine Quantification by ELISA

Cytokine levels in whole blood supernatants were quantified by enzyme-linked immunosorbent assay (ELISA). The concentrations of human IL-1β, IL-6, and TNF-α were determined as primary indicators of inflammatory activation following exposure to endotoxin-spiked hydroxyapatite discs or soluble LPS. After the incubation period, supernatants were carefully collected by centrifugation and stored at −80 °C until analysis.

ELISAs were performed using commercially available human ELISA kits (R&D Systems, Minneapolis, MN, USA; IL-1β: DY201; IL-6: DY206; TNF-α: DY210) according to the manufacturer’s instructions. Absorbance was measured at 450 nm with wavelength correction at 570 nm using a Synergy II microplate reader (BioTek Instruments, Winooski, VT, USA). Cytokine concentrations were calculated based on standard curves generated in parallel with the samples using recombinant cytokine standards provided in each kit. Standard curves were fitted using a four-parameter logistic (4PL) regression model for consistency with subsequent dose–response modelling.

For IL-1β and IL-6, supernatants obtained after overnight incubation (18–24 h) were analyzed. For TNF-α, supernatants collected after 4 h incubation were used to avoid signal saturation and ensure measurements within the dynamic range of the assay. All samples were measured in technical duplicates, and cytokine concentrations were expressed as pg/mL.

### 2.10. Multiplex Cytokine Analysis

Cytokine profiling was performed using a bead-based multiplex immunoassay (XMAP, Luminex Corp., Austin, TX, USA) to evaluate a broader inflammatory response beyond the primary MAT readouts. Plasma samples obtained after whole blood incubation with soluble LPS or LPS-associated hydroxyapatite discs were collected and stored at −80 °C until analysis. Cytokine concentrations were measured using a commercially available human 27-plex cytokine panel (Bio-Plex Pro Human Cytokine 27-plex Assay #M500KCAF0Y, Biorad, Hercules, CA, USA), according to the manufacturer’s instructions. The panel included the following analytes: GF basic (FGF basic), Eotaxin, G-CSF, GM-CSF, IFN-γ, IL-1β, IL-1ra, IL-2, IL-4, IL-5, IL-6, IL-7, IL-8, IL-9, IL-10, IL-12 (p70), IL-13, IL-15, IL-17A, IP-10, MCP-1 (MCAF), MIP-1α, MIP-1β, PDGF-BB, RANTES, TNF-α, and VEGF. Samples and standards were incubated with fluorescent capture beads, followed by detection antibodies and streptavidin–phycoerythrin conjugate. Data acquisition was performed using a Luminex S200 analyzer (Biorad, USA), and cytokine concentrations were calculated based on standard curves generated for each analyte using a five-parameter logistic (5PL) regression model (Xponent 3D, Luminex, Austin, TX, USA). Results were expressed as absolute concentrations (pg/mL). Analytes with signal values below the assay detection limit were considered not detected and excluded from graphical representation.

### 2.11. Data Analysis

All statistical analyses and nonlinear regression modelling were performed using GraphPad Prism version 8.0 (GraphPad Software, San Diego, CA, USA). Dose–response curves for IL-1β, IL-6, and TNF-α were fitted using a four-parameter logistic (4PL) regression model with variable slope, according to the equation:

*Y* = *Bottom* + (*Top* − *Bottom)*/(1 + 10^((^*^logEC^*^50 − ^*^X^*^) ∗ ^*^Hill slope^*^)^)

where Y represents cytokine concentration (pg/mL), X is the logarithm of the nominal LPS concentration (EU/mL), Top and Bottom correspond to the upper and lower asymptotes of the curve, EC50 represents the concentration producing 50% of the maximal response, and Hill slope describes the steepness of the transition between baseline and maximal response. Bottom values were left unconstrained for all analytes, and Hill slope was constrained only for IL-6, as specified in the Section 3. Goodness-of-fit was assessed using weighted R^2^ values. Nonlinear regression was performed using least squares fitting with 1/Y^2^ weighting.

Interpolated endotoxin concentrations for spike-recovery analysis were calculated by back-calculation from the fitted 4PL curves using measured cytokine concentrations obtained from hydroxyapatite-associated LPS samples. Spike recovery (%) was calculated as:

*Recovery* (%) = (*Interpolated concentration*/*Nominal concentration*) ∗ 100

A recovery range of 50–200% was considered acceptable for interpretation of endotoxin activity within the validated dynamic range of the assay. Interpolation was not performed when measured cytokine values exceeded the fitted upper asymptote (signal saturation).

For individual concentration comparisons between soluble and hydroxyapatite-associated LPS conditions, either unpaired two-tailed Welch’s or Student’s *t*-tests were applied where indicated. Statistical significance was defined as *p* < 0.05. No time-course comparisons were made. Data are presented as median values (*n* = 3 independent experiments) unless otherwise stated. Each independent experiment was performed using whole blood pooled from five healthy donors and included technical quintuplicates for spike-recovery calculations.

## 3. Results

### 3.1. Material Characterization

**Figure 1:**
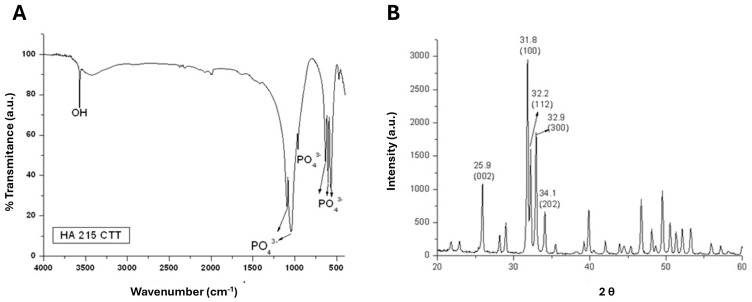
Physicochemical characterization of hydroxyapatite discs. (**A**) Fourier-transform infrared (FTIR) spectrum recorded in the range of 4000–500 cm^−1^, showing characteristic functional groups of hydroxyapatite. (**B**) X-ray diffraction (XRD) pattern collected over a 2θ range of 20–60°, confirming the crystalline phase of hydroxyapatite.

The structural identity and crystalline phase of the hydroxyapatite discs were first verified prior to biological testing. FTIR analysis (Figure 1A) showed the characteristic vibrational bands of hydroxyapatite. The broad band observed around 3500–3570 cm^−1^ corresponds to the O–H stretching vibration of structural hydroxyl groups. In the phosphate region, the intense bands between approximately 1030–1090 cm^−1^ are attributed to the ν_3_ asymmetric stretching mode of PO_4_^3−^ groups, while the band near 962 cm^−1^ corresponds to the ν_1_ symmetric stretching vibration. The doublet observed around 565 and 602 cm^−1^ is associated with the ν_4_ O–P–O bending modes, and the band near 470 cm^−1^ corresponds to the ν_2_ bending vibration. These vibrational features are characteristic of stoichiometric hydroxyapatite and confirm the structural integrity of the material prior to biological evaluation.

X-ray diffraction analysis further confirmed the crystalline structure of the material (Figure 1B). The observed diffraction peaks were compared with the standard reference pattern for hydroxyapatite (ICDD PDF No. 09-0432). The diffraction pattern exhibited well-defined peaks at 2θ values corresponding to the characteristic reflections of stoichiometric hydroxyapatite, including the (002), (211)/(112)/(300), and (202) planes. No additional peaks associated with secondary calcium phosphate phases were detected, indicating that the discs consisted predominantly of crystalline hydroxyapatite. Together, the FTIR and XRD results confirm that the material used in the subsequent experiments corresponded to hydroxyapatite with the expected chemical and crystallographic features.

### 3.2. Cytokine Evaluation

Prior to functional endotoxin assessment, preliminary experiments were conducted to exclude confounding effects related to material cytotoxicity and spiking methodology. Exposure of human monocyte-derived macrophages to hydroxyapatite extracts did not result in substantial induction of apoptosis or necrosis compared with control conditions, whereas positive controls behaved as expected (Appendix A).

In parallel, two endotoxin spiking approaches reported in the literature—immersion and dropwise application—were compared using IL-6 as functional readout. Dropwise application resulted in minimal cytokine release at both 2.5 and 5 EU, with functional recovery values of approximately 3–4% relative to immersion (Table 1).

In contrast, immersion-spiked discs produced robust IL-6 responses with substantially lower variability. Statistical comparison using two-tailed Welch’s *t*-test demonstrated significantly higher IL-6 responses following immersion-based spiking compared with dropwise application at both 2.5 EU (*p* = 0.0015) and 5.0 EU (*p* = 0.0008). Based on these findings, immersion was selected for all subsequent experiments.

**Figure 2:**
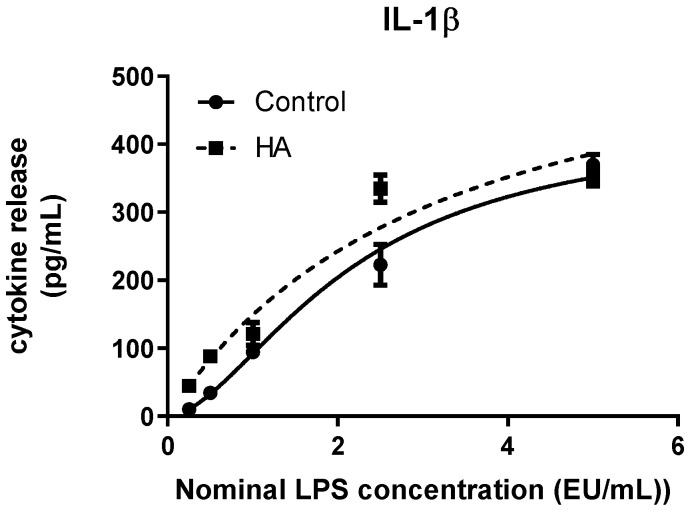
Dose–response curve of IL-1β release in whole blood following stimulation with soluble LPS (Control) or hydroxyapatite-associated LPS (HA). Cytokine concentrations were plotted against nominal LPS concentrations and fitted using a four-parameter logistic (4PL) model. Data represent median values from three independent experiments (*n* = 3), with error bars indicating interquartile range (IQR). Curve parameters are provided in Table 2.

With this approach, IL-1β release increased in a concentration-dependent manner in response to escalating LPS exposure under both experimental conditions (Figure 2). In the control condition (soluble LPS), cytokine levels rose progressively across the tested range, reaching a plateau at higher endotoxin concentrations. A similar dose–response profile was observed when LPS was associated with hydroxyapatite (HA) discs. Nonlinear regression analysis using a four-parameter logistic (4PL) model confirmed the adequacy of curve fitting for both conditions (R^2^ > 0.93; Table 2). While the overall dynamic range of the response was preserved in the presence of HA, differences in curve parameters were observed. The maximal fitted response (Top) was higher for HA-associated LPS compared with soluble LPS, whereas the estimated EC50 value was modestly shifted. Additionally, the slope of the fitted curve was reduced in the HA condition, indicating a less steep transition between baseline and maximal response.

**Figure 3:**
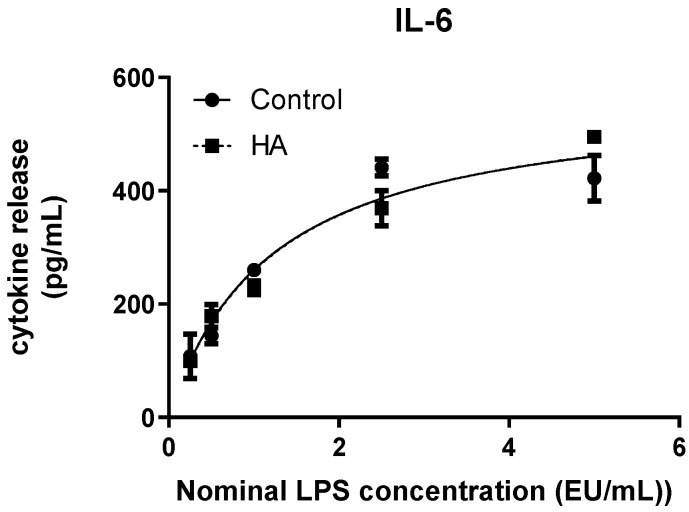
Dose–response curve of IL-6 release in whole blood following stimulation with soluble LPS (Control) or hydroxyapatite-associated LPS (HA). Cytokine concentrations were plotted against nominal LPS concentrations and fitted using a four-parameter logistic (4PL) model. Data represent median values from three independent experiments (*n* = 3), with error bars indicating interquartile range (IQR). Curve parameters are provided in Table 2.

Interleukin 6 (IL-6) release increased in a concentration-dependent manner following LPS stimulation under both experimental conditions (Figure 3). In the control condition (soluble LPS), cytokine levels rose steeply at lower endotoxin concentrations and approached a plateau at higher doses, consistent with a typical sigmoidal response profile. A comparable curve shape and dynamic range were observed when LPS was associated with hydroxyapatite (HA) discs. Four-parameter logistic (4PL) regression confirmed highly similar quantitative behaviour between conditions (Table 2). The estimated maximal response (Top) values were nearly identical for soluble LPS and HA-associated LPS, and EC50 values showed minimal variation between groups. For IL-6, the Hill slope was constrained during curve fitting, and goodness-of-fit values were high in both cases (R^2^ > 0.95), indicating robust model performance. Overall, IL-6 exhibited a reproducible and sensitive dose–response profile that was quantitatively preserved in the presence of hydroxyapatite.

**Figure 4:**
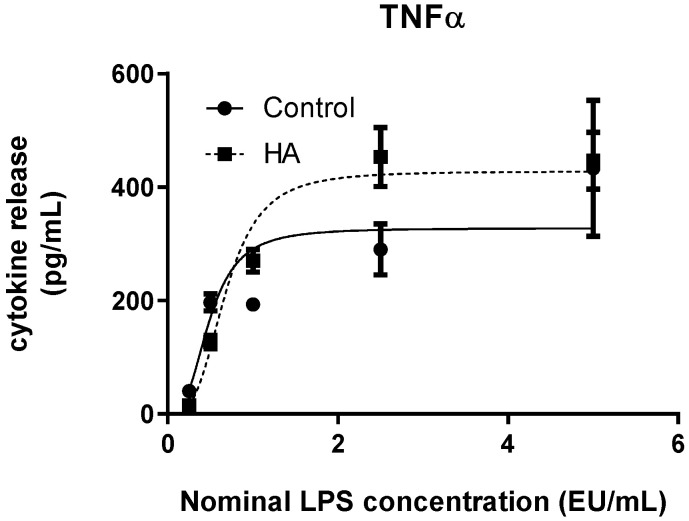
Dose–response curve of TNF-α release in whole blood following stimulation with soluble LPS (Control) or hydroxyapatite-associated LPS (HA). Cytokine concentrations were plotted against nominal LPS concentrations and fitted using a four-parameter logistic (4PL) model. Data represent median values from three independent experiments (*n* = 3), with error bars indicating interquartile range (IQR). Curve parameters are provided in Table 2.

TNF-α release increased with escalating LPS concentrations under both experimental conditions (Figure 4), displaying a steep sigmoidal dose–response profile. Compared with IL-1β and IL-6, TNF-α responses showed a more pronounced transition between baseline and maximal response levels, reflected in the higher Hill slope values obtained from 4PL fitting (Table 2). Nonlinear regression analysis indicated differences in fitted parameters between conditions. While soluble LPS reached a lower estimated maximal response (Top = 327.6 pg/mL), HA-associated LPS exhibited a higher fitted maximum (Top = 427.9 pg/mL). The estimated EC50 values were shifted toward higher concentrations in the HA condition. Goodness-of-fit values were high for both curves (R^2^ = 0.9842). At the nominal endotoxin concentration of 2.5 EU/mL, a statistically significant difference between conditions was observed (Student’s *t*-test, *p* = 0.00072), indicating condition-dependent variation in TNF-α release at elevated LPS exposure levels.

Quantitative parameters derived from 4PL modelling are summarized in Table 2. Across all analytes, curve fitting demonstrated high goodness-of-fit values (R^2^ ≥ 0.93), supporting the robustness of the regression approach. Comparison of fitted parameters highlights distinct dynamic profiles among cytokines. IL-1β and TNF-α exhibited condition-dependent differences in maximal response and EC50 estimates, whereas IL-6 displayed closely overlapping parameters between soluble LPS and HA-associated LPS. Differences in Hill slope values further underscore variations in response steepness among analytes, with TNF-α showing the most abrupt transition between baseline and maximal response.

Spike recovery analysis was performed to assess potential interference of hydroxyapatite discs with endotoxin detection in the whole blood MAT system (Table 3). Recovery was calculated as the ratio between interpolated LPS concentration and nominal LPS concentration, expressed as percentage, with an acceptance range of 50–200%. For IL-1β, recovery values were within the acceptable range at all tested concentrations except the lowest nominal dose (0.25 EU/mL), which exceeded the upper acceptance limit. At intermediate and higher concentrations, recovery values remained compliant, indicating adequate detectability of endotoxin in the presence of the material within the working range of the assay.

IL-6 exhibited recovery values consistently within the 50–200% acceptance interval across the evaluated concentration range, including the highest tested dose. These findings indicate minimal interference of hydroxyapatite with IL-6-based endotoxin quantification under the experimental conditions. For TNF-α, recovery values at lower concentrations (0.25–1.0 EU/mL) were within the acceptable range. At higher concentrations, interpolated values were not obtainable due to signal saturation above the fitted curve range, precluding recovery calculation. Overall, spike recovery analysis supports the suitability of the immersion-based spiking method for endotoxin detection in hydroxyapatite discs within the validated dynamic range of the assay.

**Figure 5:**
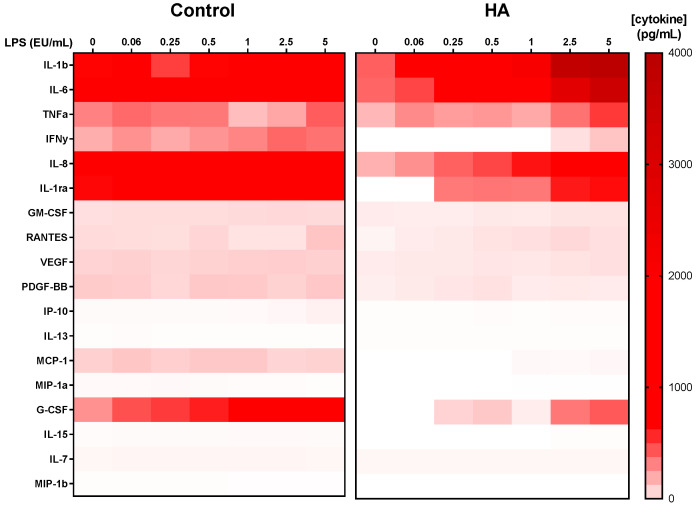
Multiplex cytokine profile in whole blood stimulated with increasing nominal LPS concentrations (0–5 EU/mL) in the absence (Control) or presence of hydroxyapatite discs (HA). Heatmap displays absolute cytokine concentrations (pg/mL), with colour intensity corresponding to concentration levels as indicated in the scale bar. Data represent median values from three independent experiments (*n* = 3). Cytokines not detected under the experimental conditions (IL-2, IL-4, IL-5, IL-9, IL-10, IL-12p70, IL-17A, and basic FGF) are not shown.

Multiplex cytokine analysis using absolute concentrations (pg/mL) confirmed that endotoxin stimulation induced a broad but selective inflammatory response in whole blood (Figure 5). Among the evaluated mediators, IL-1β, IL-6 and TNF-α were consistently among the most strongly induced cytokines across increasing LPS concentrations, together with IL-8, which also exhibited robust dose-dependent elevation. Baseline cytokine levels (0 EU/mL) in the presence of hydroxyapatite were not increased compared with control conditions and appeared slightly lower for several mediators. Upon LPS stimulation, the overall pattern of cytokine induction was preserved in the presence of the material, with the same dominant pro-inflammatory mediators observed across concentrations.

Several additional cytokines and growth-related factors showed comparatively modest or minimal changes in response to LPS, indicating that the inflammatory activation was not uniformly distributed across all analytes. Collectively, these data demonstrate that hydroxyapatite does not qualitatively alter the global cytokine response to endotoxin stimulation, while confirming that the classical MAT readouts represent some of the most responsive mediators in this system (Figure 5).

## 4. Discussion

Ceramic materials based on hydroxyapatite (HA) are widely used in dentistry as bone substitutes, implant coatings, and scaffold components due to their chemical similarity to mineralized tissue and well-established osteoconductive properties [20]. As these materials are increasingly incorporated into regenerative and implantable applications, rigorous biological safety assessment becomes essential, particularly under the framework of international biocompatibility standards [4]. Among the critical safety concerns is pyrogenicity, which may arise from endotoxin contamination introduced during manufacturing and handling processes or from adsorption and retention of bacterial components on material surfaces [21]. Surface-associated endotoxin has been shown to elicit inflammatory responses distinct from soluble LPS, highlighting the need for biologically relevant detection systems [22,23]. Ensuring reliable detection of endotoxin activity in such materials is therefore both scientifically relevant and regulatorily significant [13].

While the Monocyte Activation Test (MAT) has been validated as a functional alternative to traditional endotoxin assays, including the rabbit pyrogen test and limulus-based methods, its application to solid biomaterials presents specific methodological challenges [24,25]. In contrast to extraction-based approaches, which may underestimate surface-bound pyrogens, whole blood-based assays allow direct interaction between immune cells and the material surface, capturing the biological impact of adsorbed endotoxin [23,26]. However, ceramic substrates, particularly those with porous or adsorptive surfaces, may influence endotoxin distribution and bioavailability, thereby modulating cytokine readouts and potentially affecting assay performance [5]. The present study addressed this issue by evaluating whether a whole blood MAT platform can reliably detect endotoxin activity associated with a hydroxyapatite-based ceramic material and by systematically examining methodological factors that influence assay robustness. Notably, ceramic biomaterials have been underrepresented in prior MAT validation studies, underscoring the relevance of material-specific investigations [12]. In this context, our findings demonstrate that, when appropriate spiking methodology is employed, functional endotoxin detection is preserved in the presence of hydroxyapatite, supporting the applicability of MAT to this class of biomaterials.

The application of MAT to solid biomaterials requires careful consideration of how endotoxin is associated with the material prior to biological testing. Both dropwise application and immersion-based spiking approaches have been described, and endotoxin recovery has been shown to depend strongly on the mode of association and incubation conditions [23,26]. Surface chemistry, porosity, wettability, and adsorption capacity can influence the distribution and biological availability of endotoxin, thereby affecting functional cytokine response [6,27]. In the present study, an initial comparison between spiking strategies demonstrated that dropwise application resulted in minimal functional recovery of endotoxin activity in hydroxyapatite discs, whereas immersion-based contamination produced reproducible, dose-dependent cytokine responses within the validated dynamic range of the assay. These findings align with previous reports indicating that surface-bound endotoxin may not be uniformly accessible under static conditions and that adsorption dynamics critically influence biological detection [26].

In the context of solid biomaterials, spike recovery testing is not merely a procedural requirement but a critical indicator of assay applicability. Regulatory and methodological analyses have emphasized that material-specific interference must be excluded before interpreting cytokine output quantitatively [24,25]. Because endotoxin may adsorb to ceramic surfaces or interact with material-associated proteins, functional recovery provides insight into whether the biological readout accurately reflects nominal endotoxin exposure. Acceptable recovery within a defined range indicates that the material does not mask, potentiate, or otherwise distort cytokine-based quantification beyond the validated dynamic limits of the assay, thereby supporting the suitability of the MAT configuration for this specific hydroxyapatite substrate [12].

Within this framework, IL-1β demonstrated a consistent and well-defined dose–response behaviour in the presence of hydroxyapatite. The fitted 4PL curves revealed preservation of the overall sigmoidal profile, with high goodness-of-fit values supporting reliable modelling under both conditions. IL-1β has historically been the most frequently employed cytokine in MAT-based assays for medical devices and [25,28], and its use has been supported by extensive validation in whole blood systems [23,24]. Notably, the maximal fitted response (Top) was higher for HA-associated LPS compared with soluble LPS, while the EC50 was modestly shifted. This combination suggests that the dynamic characteristics of IL-1β release are influenced by the presence of the ceramic substrate, yet without compromising the capacity of the assay to resolve concentration-dependent differences. The reduced Hill slope observed in HA exposure further indicates a broader transition between baseline and maximal response levels, reflecting a slightly less abrupt activation profile. Such modulation is consistent with previous observations that surface-associated endotoxin may alter stimulus presentation to circulating monocytes without necessarily suppressing overall responsiveness [23,26]. Rather than indicating inhibition, this behaviour supports preserved biological reactivity accompanied by subtle modulation of cytokine kinetics or effective bioavailability at the material interface.

It should also be considered that the present study did not directly quantify the physicochemical strength of LPS adsorption to the hydroxyapatite surface nor assess residual moisture following the drying step after immersion-based spiking. Although partial desorption upon whole blood exposure cannot be completely excluded, the experimental design aimed to evaluate the functional pyrogenic response of endotoxin associated with a biomaterial surface under conditions simulating material–blood interaction. Importantly, the distinct cytokine response profiles observed between hydroxyapatite-associated and soluble LPS at equivalent nominal concentrations indicate that the biological behaviour of surface-bound endotoxin was not simply equivalent to freely soluble LPS. Even if dynamic desorption occurred, such behaviour would reflect clinically relevant surface–biomolecule interactions rather than an experimental artefact, as endotoxin bioavailability in vivo is likewise influenced by adsorption–desorption equilibria at biomaterial interfaces.

Importantly, spike recovery values remained within the predefined acceptance interval across most of the tested range, with the exception of the lowest nominal concentration. Deviations at lower concentrations are well recognized in cytokine-based MAT configurations and often reflect variability near the lower limit of quantification rather than true biological masking [25,29]. These findings indicate that IL-1β provides a functionally robust and quantitatively interpretable readout for endotoxin activity in hydroxyapatite discs when immersion-based spiking is employed.

In contrast to IL-1β, IL-6 exhibited highly consistent dynamic behaviour in the presence and absence of hydroxyapatite. The fitted 4PL curves were nearly superimposable, with closely matching Top and EC50 values between soluble and HA-associated LPS. Although IL-6 is less frequently used as a primary MAT endpoint compared with IL-1β [25], it has been shown to provide stable and sensitive detection of endotoxin-induced activation in whole blood systems [30]. Furthermore, IL-6 levels are associated with successful bone regeneration outcomes in dental implantology and biomaterial integration [31], highlighting its clinical relevance beyond pyrogen detection. The near overlap observed here indicates that endotoxin activity remained quantitatively comparable across conditions, suggesting minimal influence of the ceramic substrate on overall magnitude or sensitivity of the IL-6 response. The Hill slope was constrained during curve fitting, reflecting the regularity and predictability of the sigmoidal transition. Even under this modelling constraint, goodness-of-fit values were high in both conditions, reinforcing the analytical robustness of IL-6 in this system. Unlike IL-1β, no deviation outside the predefined recovery acceptance range was observed across the tested concentrations, and interpolated values remained within the dynamic window of the assay. In the context of assay harmonization and readout selection, an issue repeatedly highlighted in methodological analyses of MAT [13,25], these findings position IL-6 as the most analytically stable cytokine among those evaluated for hydroxyapatite discs. The preservation of dynamic range, slope behaviour, and recovery performance suggests that IL-6 may offer particular advantages when evaluating ceramic biomaterials, where surface interactions could otherwise modulate stimulus kinetics.

TNF-α displayed a distinct response profile compared with IL-1β and IL-6. Although a clear dose–response relationship was observed under both conditions, the fitted curves exhibited markedly steeper slopes, indicating a more abrupt transition between baseline and maximal cytokine release. TNF-α is known to be an early and rapidly amplified mediator in endotoxin-driven inflammatory cascades, often displaying sharper dynamic transitions compared with downstream cytokines such as IL-6 [32]. In the presence of hydroxyapatite, the estimated maximal response was higher and the EC50 shifted relative to soluble LPS, reflecting altered dynamic characteristics rather than loss of responsiveness. These differences may indicate greater sensitivity of TNF-α kinetics to variations in stimulus presentation or microenvironmental factors at the material interface, consistent with reports that TNF-α responses can be more susceptible to assay configuration and matrix effects in complex systems [25,28]. However, practical limitations were also evident. At higher endotoxin concentrations, TNF-α responses approached signal saturation, restricting reliable interpolation for spike-recovery analysis within the upper range of the assay. This narrower usable window contrasts with the broader dynamic performance observed for IL-6 and, to a lesser extent, IL-1β. Such behaviour underscores the importance of carefully defining the analytical range when TNF-α is employed as a primary readout in solid biomaterial testing. Based on the present data, a narrower working range within the lower-to-intermediate endotoxin concentrations (≤1 EU/mL under the current assay configuration) is recommended when TNF-α is selected as the primary endpoint, in order to avoid signal saturation and ensure reliable quantitative interpolation. In line with previous observations in MAT applications for medical devices, TNF-α may serve as a complementary rather than standalone endpoint, particularly when quantitative interpolation and recovery analysis are required [13].

Beyond the primary MAT readouts, multiplex cytokine profiling provided a broader view of the inflammatory landscape induced by endotoxin in the presence of hydroxyapatite. Whole blood stimulation by LPS is known to trigger a coordinated cytokine cascade driven primarily through TLR4-mediated activation of monocytes, resulting in early TNF-α release followed by induction of IL-1β, IL-6, and chemokines such as IL-8 [33]. Analysis of absolute cytokine concentrations confirmed that IL-1β, IL-6, and TNF-α ranked among the most strongly induced mediators across increasing LPS concentrations, together with IL-8. This pattern reinforces the biological relevance of the classical MAT analytes within the wider cytokine network activated in whole blood and is consistent with previous descriptions of endotoxin-driven cytokine hierarchies in MAT systems [25,28]. Importantly, baseline cytokine levels in the presence of hydroxyapatite (0 EU/mL) were not elevated compared with control conditions and were, in several instances, slightly lower. This observation supports the absence of intrinsic pro-inflammatory activity of the ceramic substrate under the experimental configuration used, consistent with reports that hydroxyapatite elicits minimal cytokine production from blood monocytes/macrophages in the absence of additional stimuli [34].

In the context of device evaluation, exclusion of nonspecific activation is a critical prerequisite before interpreting endotoxin-specific responses [13,24]. The absence of elevated baseline mediator release therefore strengthens the conclusion that observed cytokine induction reflects endotoxin activity rather than material-driven immune stimulation. While the overall hierarchy of LPS-induced mediators was preserved between soluble and HA-associated conditions, quantitative differences were observed for selected cytokines. For example, pro-inflammatory mediators such as IL-1β and IL-6 remained prominently induced, whereas certain growth-related or regulatory factors, including VEGF and IL-7, displayed comparatively modest responses. Differential modulation of secondary mediators may reflect variations in stimulus presentation or local microenvironmental effects at the material–blood interface, without fundamentally altering the dominant inflammatory signature [21]. Such subtle shifts are compatible with the concept that surface-associated endotoxin can modulate response amplitude without disrupting the overall cytokine cascade [23,26]. Taken together, the multiplex data situate the classical MAT cytokines within a coherent inflammatory response profile that remains qualitatively intact in the presence of hydroxyapatite. Rather than introducing aberrant immune activation, the ceramic substrate appears to preserve the canonical LPS-induced cytokine architecture, supporting the biological validity of the assay configuration for this material class.

The present findings have practical implications for the biological safety assessment of ceramic biomaterials used in bioengineering and dental applications. Hydroxyapatite-based ceramics are widely employed as bone graft substitutes, coatings, and regenerative scaffolds, where surface area, porosity, and adsorption properties are intrinsic to their clinical function [35]. These same characteristics may influence endotoxin association and biological detection, underscoring the importance of material-specific validation when implementing functional pyrogen testing strategies [24,26]. Our data indicate that, for this hydroxyapatite material, immersion-based endotoxin spiking is required to ensure reliable functional detection in a whole blood MAT system. In addition, comparative analysis of cytokine readouts revealed differential analytical performance across endpoints, with IL-6 exhibiting the most stable quantitative behaviour and TNF-α displaying a narrower usable dynamic range. Such endpoint-dependent variability has been previously discussed in methodological evaluations of MAT implementation [13,25].

Recent efforts to standardize in vitro pyrogen testing for medical devices have further emphasized the importance of adapting MAT configurations to the specific characteristics of solid materials. The development of device-oriented MAT frameworks has highlighted the need for material-specific validation strategies to ensure reliable detection of surface-associated pyrogens while reducing reliance on animal-based assays. Studies evaluating MAT implementation in the context of solid medical devices have demonstrated that assay configuration, extraction procedures, and surface interactions critically influence analytical performance, reinforcing the necessity of tailored methodological approaches for different material classes [12,13,25]. These broader standardization initiatives align with the present findings, which underscore that hydroxyapatite ceramics require optimized spiking strategies and endpoint selection to achieve robust functional detection.

It should be emphasized that immersion-based spiking, as implemented in the present study, is not intended to replicate a specific industrial contamination scenario. In real-world manufacturing and storage settings, endotoxin contamination may occur through multiple routes, including airborne particulate deposition, contact with contaminated surfaces, or exposure to aqueous environments during processing. Localized surface contamination may therefore more closely resemble dropwise deposition in certain contexts. However, the purpose of immersion-based spiking in the present investigation was methodological rather than environmental simulation. This approach ensures more homogeneous association of endotoxin with the material surface and allows standardized assessment of functional recovery and assay performance. For analytical validation of MAT in solid biomaterials, reproducibility and controlled surface association are essential prerequisites. Thus, while immersion-based spiking does not represent a single real-world contamination pathway, it provides a robust and conservative model for evaluating endotoxin detectability under conditions of sustained surface association.

While these observations collectively support a tailored approach to MAT application in dental ceramics, several considerations should be taken into account when interpreting them. Spike-recovery analyses were derived from three independent experimental runs, each performed with technical quintuplicates and using whole blood pooled from five healthy donors. Pooling strategies are commonly employed in MAT configurations to mitigate inter-individual variability and enhance analytical stability [25]. However, while pooling strengthens robustness within the assay configuration employed, it does not fully address broader sources of variability that may emerge across laboratories, donor populations, or blood preparation workflows. In addition, the investigation was conducted using a single hydroxyapatite-based ceramic material in a defined disc configuration. Although representative of a clinically relevant class of dental biomaterials, the results cannot be directly generalized to all hydroxyapatite formulations or to other calcium phosphate ceramics with distinct surface characteristics, porosity profiles, or manufacturing parameters [36]. Importantly, although the MAT offers the recognized advantage of detecting both endotoxin and non-endotoxin pyrogens, including bacterial lipoproteins and lipoteichoic acid (LTA) [13,25], the present investigation focused exclusively on LPS as a controlled model stimulus. This approach allowed the systematic evaluation of material-associated interference and quantitative assay performance under defined conditions. Future investigations incorporating additional pyrogen classes may further clarify whether surface–stimulus interactions differ according to molecular structure, receptor engagement pathways (e.g., TLR2 versus TLR4), or adsorption behaviour on ceramic substrates.

The comparison between spiking methodologies was performed as a targeted evaluation using IL-6 as the functional readout to guide method selection for this material. While sufficient to demonstrate the necessity of immersion-based spiking in this context, broader analyte comparisons may further refine protocol recommendations for other ceramic formats. Finally, TNF-α responses exhibited signal saturation at higher endotoxin concentrations, limiting interpolation in the upper dynamic range and underscoring the importance of concentration-range optimization when selecting primary endpoints. Taken together, the present study provides a structured analytical framework for MAT application in hydroxyapatite ceramics and may serve as a foundation for future interlaboratory validation efforts aimed at establishing standardized approaches for endotoxin activity testing in solid dental biomaterials.

## 5. Conclusions

In summary, this study demonstrates that whole blood MAT can be reliably applied to a hydroxyapatite-based ceramic when immersion-based endotoxin association is employed. Endotoxin activity remained functionally detectable in the presence of the material, and 4PL modelling enabled quantitative comparison of cytokine responses under both conditions. IL-6 emerged as the most analytically stable readout, while IL-1β and TNF-α provided complementary information within their validated ranges. Multiplex analysis confirmed the preservation of the overall inflammatory signature and the absence of baseline activation by the ceramic substrate. These findings support the use of MAT for endotoxin activity assessment in hydroxyapatite-based dental biomaterials, provided that material-specific methodological optimization is implemented.

## Figures and Tables

**Table 1:** IL-6 response following dropwise endotoxin spiking compared with immersion spiking.

Spiking Method	LPS (EU)	Mean IL-6 (pg/mL)	SD (pg/mL)	CV (%)	Functional Recovery (%) †
Dropwise (disc in plate)	2.5	16.506	13.847	83.9	3.13
Immersion (disc in tube)	2.5	285.413 *	80.080	28.1	100 *
Dropwise (disc in plate)	5.0	21.145	14.979	70.8	4.26
Immersion (disc in tube)	5.0	382.794 *	93.549	24.4	100 *

† Functional recovery (%) calculated relative to immersion-spiked discs at the same nominal LPS concentration. Immersion values used as reference (set to 100%). Statistical comparisons were performed using two-tailed Welch’s *t*-tests. * *p* < 0.01 vs. dropwise at the same nominal LPS concentration (*n* = 5).

**Table 2:** 4PL dose–response curve parameters (Control vs. HA).

Analyte	Condition	Bottom (pg/mL)	Top (pg/mL)	EC50 (EU/mL)	logEC50	Hill Slope	R^2^ (Weighted)
IL-1β	Control	0.000	431.3	2.129	0.328	1.727	0.973
IL-1β	HA	0.000	622.8	3.101	0.492	1.022	0.937
IL-6	Control	0.000	569.9	1.188	0.075	1.000 *	0.956
IL-6	HA	0.000	572.5	1.208	0.082	1.000 *	0.979
TNF-α	Control	0.000	327.6	0.489	−0.311	2.865	0.984
TNF-α	HA	0.000	427.9	0.682	−0.167	3.263	0.984

Nonlinear regression (4PL, variable slope). Bottom unconstrained for all analytes. Hill slope constrained only for IL-6 (*).

**Table 3:** Spike Recovery Analysis.

Analyte	Nominal LPS (EU/mL)	Interpolated LPS (EU/mL)	Recovery (%)	Within 50–200% Range
IL-1β	0.3	0.6	244	No
IL-1β	0.5	1.0	194	Yes
IL-1β	1.0	1.2	124	Yes
IL-1β	2.5	4.4	175	Yes
IL-1β	5.0	5.0	100	Yes
IL-6	0.3	0.3	101	Yes
IL-6	0.5	0.5	109	Yes
IL-6	1.0	0.8	80	Yes
IL-6	2.5	2.2	88	Yes
IL-6	5.0	7.9 *	157	Yes
TNF-α	0.3	0.2	68	Yes
TNF-α	0.5	0.4	83	Yes
TNF-α	1.0	0.8	84	Yes
TNF-α	2.5	**	**	N/A
TNF-α	5.0	**	**	N/A

Recovery (%) calculated as: (Interpolated LPS/Nominal LPS) × 100. Acceptance range: 50–200% (according to MAT guideline practice). * Extrapolated values not within curve range. ** Not interpolable (above Top). N/A: Not Applicable.

**Figure A1:**
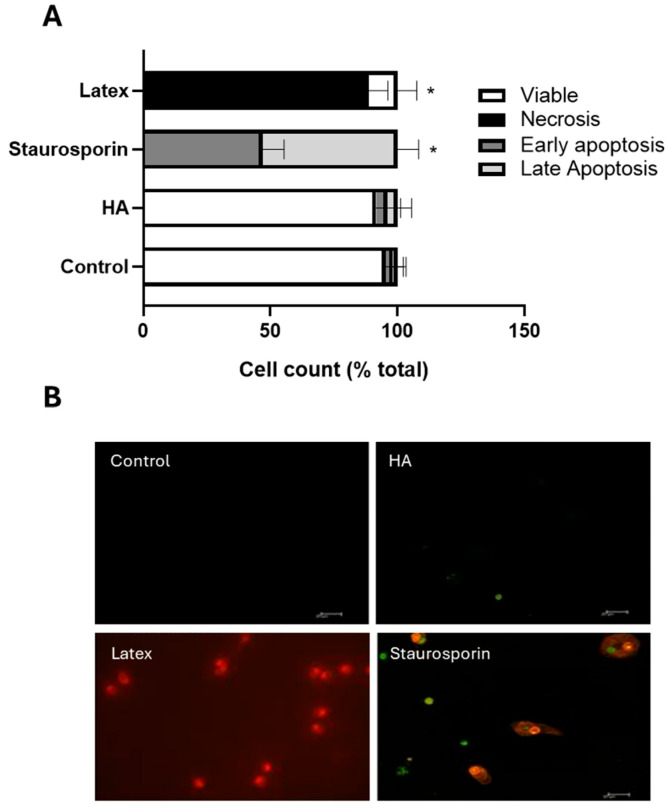
In vitro assessment of apoptosis and necrosis following exposure to HA extracts. (**A**) Quantification of viable, early apoptotic, late apoptotic, and necrotic cells after 24 h exposure to hydroxyapatite (HA) disc extracts prepared at a surface area-to-volume ratio of 6 cm^2^/mL, according to ISO 10993-12. Latex extract and staurosporine were included as positive controls for necrosis and apoptosis, respectively. Data represent two independent experiments performed in quintuplicate coverslips per condition. * significant difference from other groups (*p* < 0.05). (**B**) Representative fluorescence micrographs of Annexin V-FITC/propidium iodide (PI) staining after 24 h exposure to material extracts. Annexin V-FITC–positive cells (green) indicate early apoptotic cells; PI-positive cells (red) indicate loss of membrane integrity (necrotic cells); double-positive cells (green/red overlay) correspond to late apoptotic cells; unstained cells are considered viable. HA extracts were prepared at a surface area-to-volume ratio of 6 cm^2^/mL (ISO 10993-12), and latex extract was prepared at 200 mg/mL. Scale bars indicate 20 μm.

## Data Availability

The original contributions presented in this study are included in the article; further inquiries can be directed to the corresponding author.
